# Electric Scooter Injuries in Tampa, Florida, Are Associated With High Rates of Head Injury, Hospital Admission, and Emergency Medical Service Transport and Low Rates of Helmet Use

**DOI:** 10.7759/cureus.39523

**Published:** 2023-05-26

**Authors:** Theo Sher, Jay Shah, Emily A Holbrook, Andrew Thomas, Jason Wilson

**Affiliations:** 1 Emergency Medicine, University of South Florida Morsani College of Medicine, Tampa, USA; 2 Anthropology, University of South Florida, Tampa, USA

**Keywords:** motor vehicle collision, ems, motor vehicle accident, helmet use, head injury, emergency medical services, e-scooter injury, electric scooter injury, research in emergency medicine, electric scooter accidents

## Abstract

Background

Standing electric scooters (e-scooters) were introduced in Tampa, Florida, in 2019. We reviewed 292 e-scooter injury cases at the Tampa General Hospital Emergency Department (ED) to determine what insights we could gain. We sought to identify the characteristics of such presentations, including chief complaint (CC), patient age, day of the week, time of day, length of stay, disposition, acuity, and means of arrival to the ED. We were particularly interested in studying the rates of hospital admission, Emergency Medical Service (EMS) transport, emergent acuity presentations, and head injuries. We also sought to identify the prevalence of alcohol use prior to e-scooter accidents and its effect on the above factors.

Methodology

This was a retrospective chart review and was exempt from the University of South Florida’s Institutional Review Board approval (STUDY004031). Data from routine clinical care in the Tampa General Hospital ED, a Level-1 Trauma Center ED in Tampa, Florida, from July 19, 2019, to May 30, 2022, were collected through an operational report within the business intelligence infrastructure of the hospital’s electronic medical record system. Data from patients with scooter injury-related encounter codes were extracted to an electronic data capture form and deidentified.

Narratives were reviewed to exclude uncertain cases (e.g., patients with moped, kick scooter, mobility scooter injuries, etc.) and to flag for alcohol endorsement, altered mental status, helmet usage, and head injuries that were not listed as the CC. CC, means of arrival, acuity, disposition, arrival/departure day of week, and arrival/departure hour were collected. Data analysis was completed using Microsoft Excel version 16.5 (Microsoft Corp., Redmond, WA, USA) and SPSS Statistics version 28.0 (IBM Corp., Armonk, NY, USA).

Results

A total of 292 of 442 collected cases remained after removing irrelevant flags. Overall, 30.8% (n = 90) of patients were between the ages of 21 and 30, and most patients presented on weekends and nights. Moreover, 40.8% (n = 119) suffered head injuries, 40.8% (n = 119) arrived via EMS, 31.5% (n = 92) were admitted to the hospital, and 18.8% (n = 55) were designated as emergent acuity. Apart from the admission rate, these rates were all higher among alcohol endorsers (39, 13.4%) than non-endorsers (253, 86.6%). Only 2.1% of patients endorsed helmet use.

Conclusions

We found higher rates of hospital admission and EMS transports in our ED than many previous studies in urban areas have reported. Our data suggest that alcohol use increases the risk of more serious e-scooter injuries, characterized by higher acuity, EMS transport rate, and head injuries among alcohol endorsers. These findings are highly relevant due to the rapidly growing e-scooter presence across the United States and may serve to inform hospitals and EMS systems regarding their role in injury management, as well as future policy regarding their safe use.

## Introduction

In 2019, a bill went into effect allowing micro-mobility devices such as shared standing electric scooters (e-scooters) to be used in bike lanes and roads in the state of Florida [1]. At the end of May 2019, the city of Tampa launched a pilot program with four of the nation’s largest providers of shared micro-mobility devices to service the city [2]. Since their rapid distribution, Tampa has seen a sharp increase in patients presenting to the Emergency Department (ED) with e-scooter-related accidents.

E-scooters were first introduced to the United States on a large scale in 2017 in the cities of San Francisco, Los Angeles, and Washington DC [3]. They have since seen a major global eruption, estimated to be a $20.78 billion market in 2021 [4]. Researchers from EDs in cities across the world have published case series regarding injuries associated with e-scooters, although the literature on the topic is still relatively incomplete and inconsistent [5].

Even before their widespread implementation in 2017, there were a significant number of e-scooter-related injuries in the United States, with Aizpuru et al. (2019) reporting 32,400 injuries in the National Electronic Injury Surveillance System (NEISS) from 2013 to 2017 [6]. Several studies have reported on the prevalence of e-scooter accidents in EDs before and after the introduction of a rental program in their cities, showing a major uptick in cases post-introduction [7-9]. Still, many lawmakers and shared e-scooter companies have not prioritized the enforcement of uniform safety precautions among riders, and highly variable degrees of regulation exist in different geographic areas [10].

Multiple studies have reported that head injuries are particularly common in such accidents [3,5-8,10-12], and helmet use has been found to be low [5,7,8,11-14]. Studies have reported varying types of injuries and degrees of severity, although severe injuries requiring hospital admission are not uncommon [3,5-8,11,13]. A systematic review of studies on e-scooter injuries by Toofany et al. (2021) reported that head, upper extremity, and lower extremity injuries were the most common [5]. Alcohol intoxication before e-scooter accidents has been reported in several studies [5,7,8,11,13-15] and was in some cases associated with more severe injury [13,15]. Bicyclist accidents have been better characterized over time, and alcohol has been associated with increased injury severity, mortality, and hospital resource utilization [16-18].

The current study aims to add to the literature on e-scooter accidents and provide further insights into their nature. To our knowledge, relevant studies out of Level 1 Trauma Centers in major cities in the United States are limited in number. Tampa is a rapidly growing city with lenient policies on e-scooters, and we believe it is imperative to study their associated injuries to help inform hospital systems, Emergency Medical Service (EMS) systems, and policymakers.

This article was previously presented as a meeting abstract at the 2023 American Academy of Emergency Medicine Annual Scientific Assembly on April 23rd, 2023, and at the 2022 Florida College of Emergency Physicians’ Symposium by the Sea on August 5th, 2022.

## Materials and methods

This was a retrospective chart review and was exempt from the University of South Florida’s Institutional Review Board approval (STUDY004031). Data from routine clinical care in a Level-1 Trauma Center ED in Tampa, Florida, from July 19, 2019, to May 30, 2022, were collected through an operational report within the business intelligence infrastructure of the hospital’s electronic medical record (EMR) system. Data from 442 patients with scooter injury-related encounter codes were extracted to an electronic data capture form and deidentified. The collected data included ED arrival and departure date and hour, patient age, chief complaint (CC), ED narrative, means of arrival to the ED, ED disposition, and patient acuity at ED intake.

ED narratives of these patient encounters were manually and independently reviewed by two coauthors (TS and JS) to exclude uncertain or irrelevant cases (e.g., patients with moped, kick scooter, mobility scooter injuries, etc.), yielding 292 relevant encounters for analysis. Any disagreement between the authors was resolved in favor of excluding the case. From the narrative information, flags were created for alcohol intoxication, altered mental status, helmet usage, and head injuries which were not listed as the CC. Head injuries listed as the CC were also flagged. Altered mental status was flagged if narratives mentioned descriptors such as disorientation, confusion, memory impairment, or active intoxication. If any flagging criteria were not mentioned in the narrative or CC, they were listed as unknown. CC, means of arrival, acuity, disposition, arrival and departure date, and arrival and departure hour were reviewed and coded for analysis.

Descriptive analysis and figures were completed in Microsoft Excel version 16.5 (Microsoft Corp., Redmond, WA, USA). Patient percentages were calculated by dividing the patient count by the total number of relevant encounters in the study. To determine injuries over time, patient number within each fiscal quarter during the study period was counted. Patient ages were divided into increments of 10 or otherwise categorized as unknown. The arrival day of week was extrapolated from the arrival date for day-of-week analysis. Arrival time was rounded to the nearest hour for visualization purposes. Length of stay was calculated as the difference between the exact departure and arrival times. For means of arrival, any ambulance arrival code was recategorized into “EMS” and any other known means of arrival (i.e., car, walk-in, or wheelchair) was recategorized into “walk-in.”

Statistical analysis was conducted with SPSS Statistics version 28.0 (IBM Corp., Armonk, NY, USA). Patient demographic and diagnostic information was entered and categorized based on quality (nominal) and used to generate descriptive statistics to identify variable relationships. Relationships were evaluated among encounters flagged for alcohol intoxication, head injuries, altered mental status, and helmet use. The relationships between alcohol intoxication and admission rate, altered mental status rate, emergent acuity rate, EMS transport rate, head injury rate, and helmet use rate were also evaluated to determine whether alcohol use increases these risks. Further, 95% confidence intervals (n = 292) and p-values (p < 0.05) were used to determine statistical significance when independently appropriate.

## Results

Injuries over time

**Figure 1:**
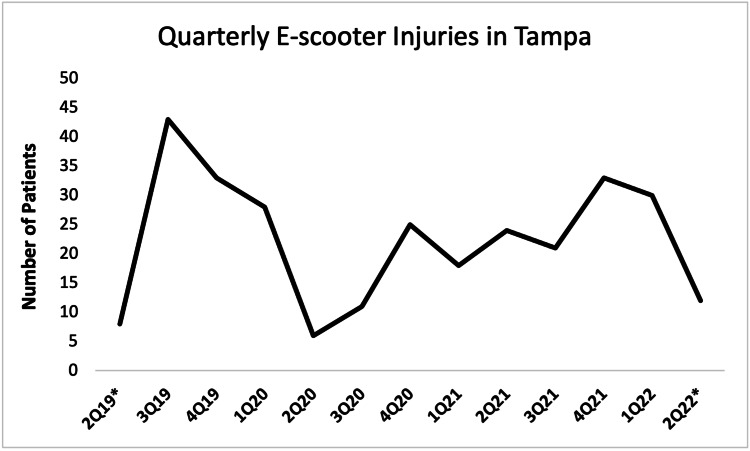
Quarter-on-quarter e-scooter injuries during the study period. *: Incomplete data period.

A total of 292 of 442 collected cases remained after excluding irrelevant or uncertain cases. We observed most patients (43, 14.7%) during the third quarter of 2019, which was the first complete quarter after the launch of the e-scooter pilot program in Tampa (Figure 1). E-scooter injuries reached a low in the second quarter of 2020, likely due to closures associated with the COVID-19 pandemic. Rates of injuries appear to be steadily rising since.

CC and flags

**Figure 2:**
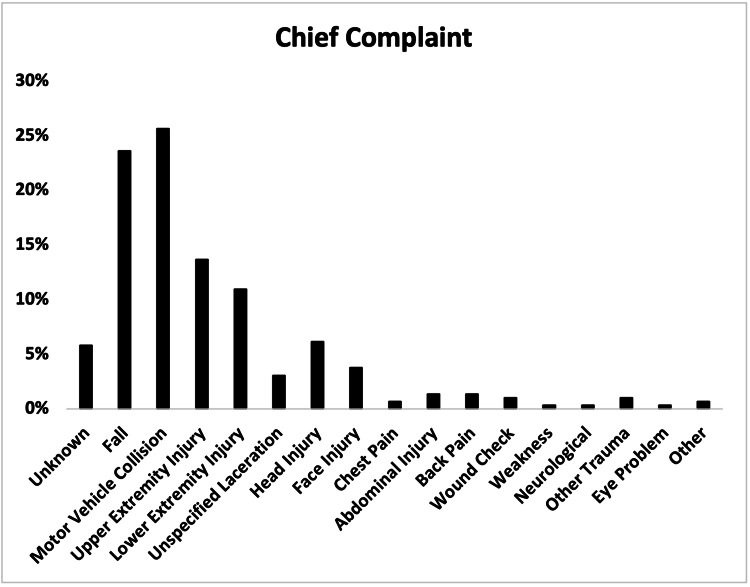
Chief complaints of patients with e-scooter injuries.

The most common chief complaint was motor vehicle collision (MVC) (75, 25.7%), followed by fall (69, 23.6%), upper extremity injury (40, 13.7%), and lower extremity injury (32, 11.0%) (Figure 2).

**Figure 3:**
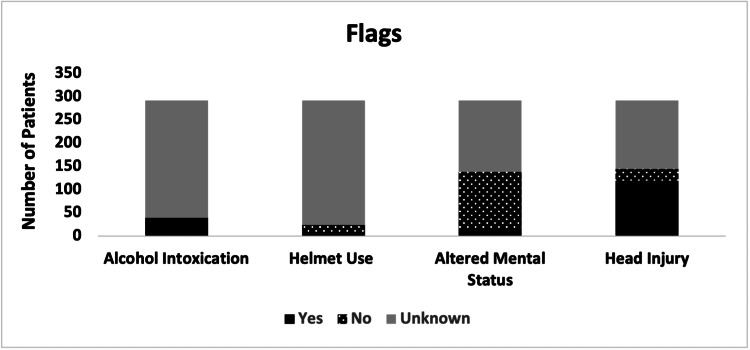
Flags for alcohol intoxication, helmet use, altered mental status, and head injuries among patients with e-scooter injuries.

Based on subjective narrative data, 39 (13.4%) out of 292 patients endorsed alcohol use, 119 (40.8%) suffered from head injuries, 14 (4.8%) presented with an altered mental status, and six (2.1%) endorsed wearing helmets (Figure 3).

Patient characteristics

**Figure 4:**
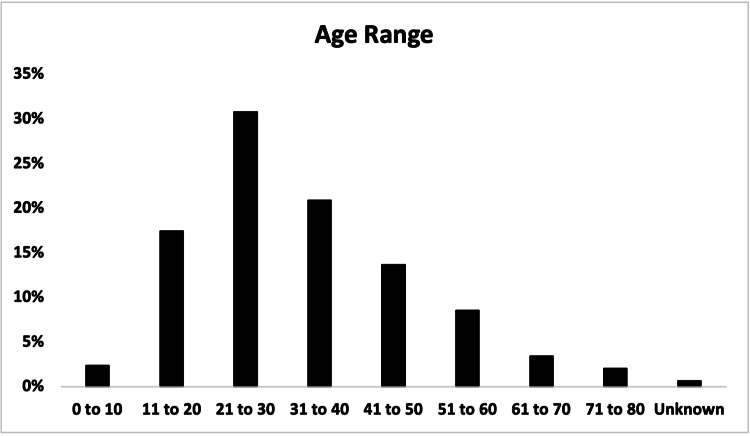
Age distribution of patients presenting with e-scooter injuries.

Young adults were the most commonly presenting age group, with 90 (30.8%) out of 292 patients being between the ages of 21 and 30 (Figure 4). In total, 61 (20.9%) were between the ages of 31 and 40, and 81 (27.7%) were above the age of 40. Patients over the age of 40 were also 8.3% more likely to be admitted to the hospital.

**Figure 5:**
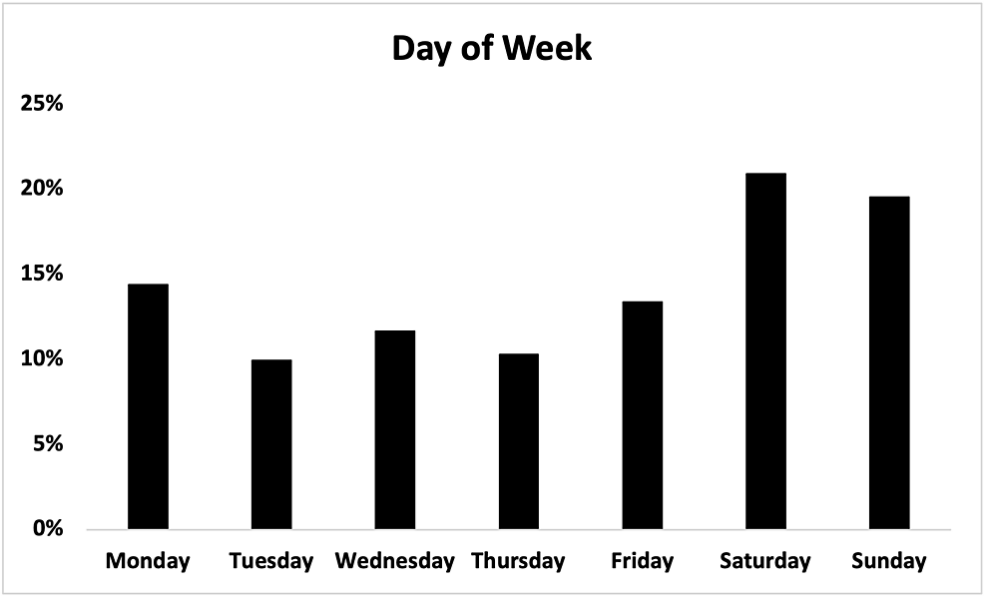
Day of week distribution of patients presenting with e-scooter injuries.

**Figure 6:**
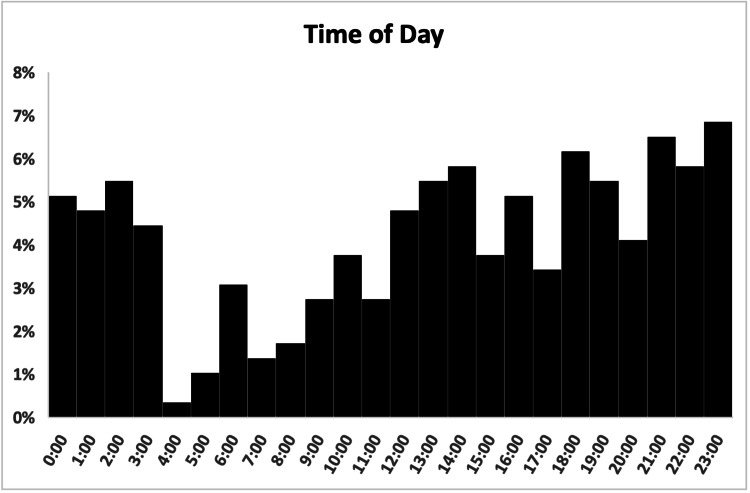
Time of day distribution of patients presenting with e-scooter injuries.

Patients most commonly presented on the weekend (Saturday and Sunday) (118, 40.4%) and between 6 pm and 4 am (161, 55.1%) (Figures 5, 6).

**Figure 7:**
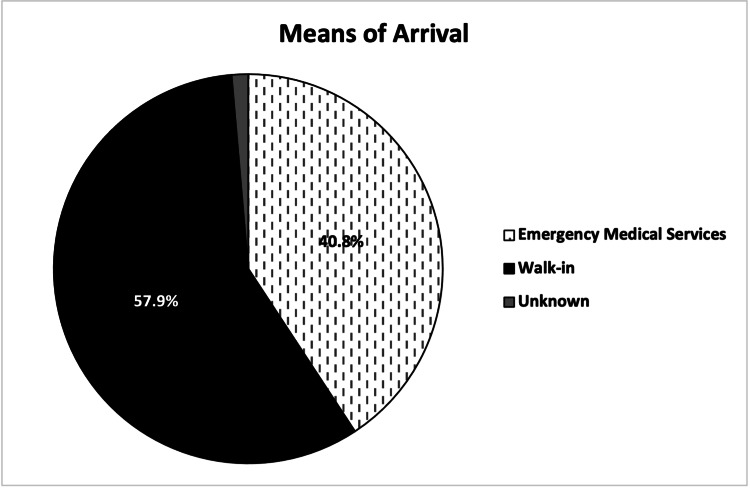
Means of arrival of patients presenting with e-scooter injuries.

**Figure 8:**
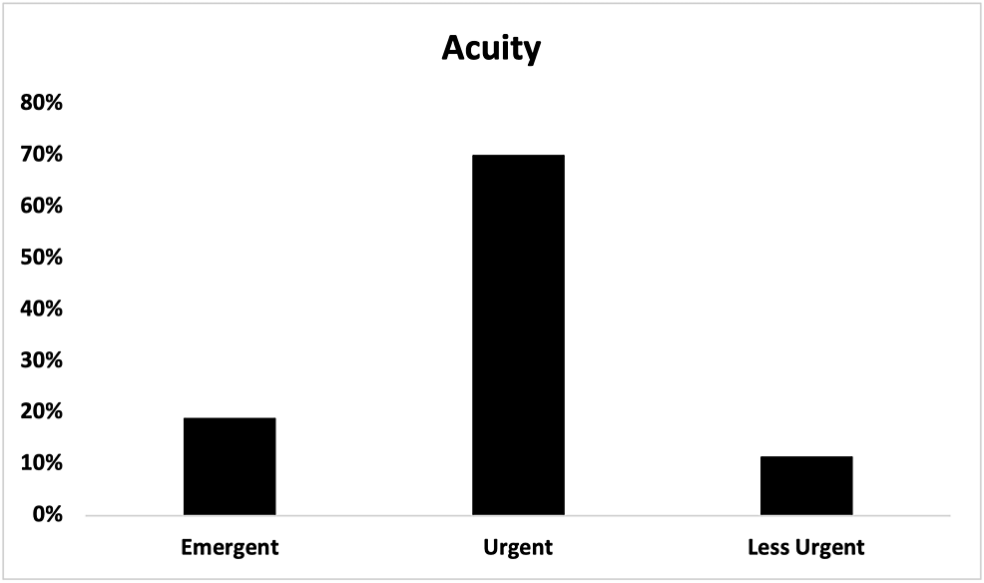
Acuity at Emergency Department intake of patients presenting with e-scooter injuries.

**Figure 9:**
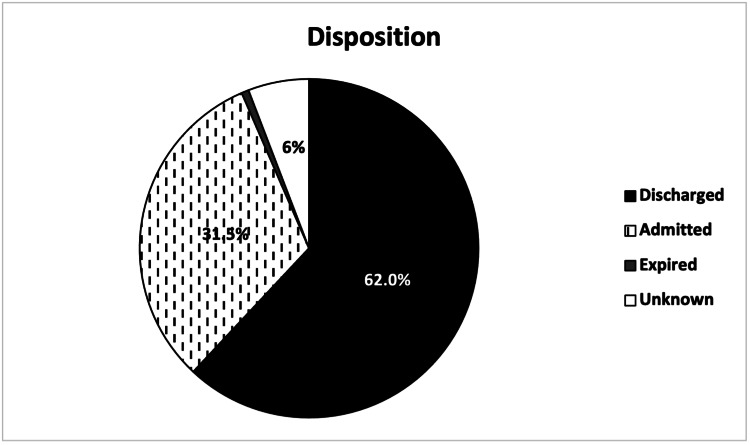
Disposition of patients presenting with e-scooter injuries.

A total of 169 (57.9%) out of 292 patients arrived at the ED via walk-in, and 119 (40.8%) arrived via EMS, including two (0.7%) via air ambulance (Figure 7). In total, 204 (69.9%) patients were designated as urgent acuity at ED intake, followed by 55 (18.8%) designated as emergent (Figure 8). Patients were most commonly discharged from the ED (181, 62.0%) or admitted to the hospital (92, 31.5%) (Figure 9). Two (0.7%) patients expired due to their injuries. The average length of stay in the ED was 5.4 hours (range = 0.6, 21.9, SD = 0.1), and was greater in patients admitted to the hospital (mean = 7.0, range = 1.4, 21.9, SD = 3.9) than patients who were discharged (mean = 4.7, range = 0.6, 14.4, SD = 2.4).

Relationships

Self-reported alcohol use was significantly correlated with head injury (r = 0.248, p < 0.001), and alcohol endorsers were twice as likely to suffer a head injury, with head injuries reported in 28 (71.7%) out of 39 endorsers and 91 (36.0%) out of 253 non-endorsers (Table 1). Alcohol endorsers had a 24.0% higher rate of arrival via EMS (24, 61.5%) than non-endorsers (95, 37.5%) (r = 0.163, p = 0.05). Upon ED intake, alcohol endorsers were more than twice as likely to be designated as having an emergent acuity (15, 38.5%) than non-endorsers (40, 15.8%) (r = 0.197, p < 0.001). There was a significant correlation between alcohol use and altered mental status (r = 0.242, p < 0.001). There were similar admission rates among alcohol endorsers (12, 30.8%) and non-endorsers (80, 31.6%) (r = -0.006, p = 0.915). Of patients who endorsed alcohol use, none reported wearing a helmet, although the sample size of helmet endorsers was low (n = 6). The chi-squared (χ^2^) test conducted at the 95th percentile and one degree of freedom confirmed the statistical relevance of these findings as head injuries (χ^2^ = 17.963), EMS transport rate (χ^2^ = 7.771), emergent acuity (χ^2^ = 11.341), and altered mental status (χ^2^ = 17.063) all had χ^2^ statistics greater than the appropriate χ^2^_crit_ of 3.841.

**Table 1:** Comparison of risk factors for alcohol endorsers versus non-endorsers. *: Indicates statistically significant relationship based on chi-squared critical value for 1 degree of freedom at 95th percentile (χ^2^_crit_ = 3.841). **: Indicates statistically significant relationship at the 95th percentile (p ≤ 0.05). χ^2 ^= chi-squared statistic; df = degrees of freedom

	Alcohol endorsers (n = 39)	Alcohol non-endorsers (n = 253)	χ^2^ tatistic (df = 1)	P-value	Correlation coefficient
Admission rate	30.8% (n = 12)	31.6% (n = 80)	0.011	0.915	-0.006
Altered mental status rate	17.9% (n = 7)	2.8% (n = 7)	17.063*	<0.001**	0.242
Emergent acuity rate	38.5% (n = 15)	15.8% (n = 40)	11.341*	<0.001**	0.197
EMS transport rate	61.5% (n = 24)	37.5% (n = 95)	7.771*	0.05**	0.163
Head injury rate	71.8% (n = 28)	36.0% (n = 91)	17.963*	<0.001**	0.248
Helmet use rate	0.0% (n = 0)	2.4% (n = 6)	0.944	0.331	-0.057

## Discussion

Since the global introduction of shared e-scooters, concerns have grown regarding their safety, and a growing body of literature has identified thousands of e-scooter-related injuries [3,5,7-15,19-21]. We have identified a large number of injuries associated with e-scooter accidents since the introduction of a shared rental program in Tampa, Florida (Figure 1). The total number of e-scooter injuries in our city is likely much higher, as patients were sampled from only one ED, and we suspect that many patients present to urgent care and primary care clinics based on news reports around the country [22,23].

The majority of patients in this study (151, 51.7%) were between the ages of 21 and 40 (Figure 4), which is consistent with other studies from after the introduction of shared e-scooter programs [3,8,12-15]. This is a shift from school-age children and adolescents being the main at-risk demographic before these programs [6]. Still, 58 (19.9%) patients were between the ages of 0 and 20, and 81 (27.7%) were above the age of 40, indicating that younger and older populations are still at significant risk. Care should be taken not to stigmatize e-scooter injuries as a primarily Millennial or Generation Z phenomenon to best support patients of all age groups, especially considering a higher proportion of admissions (8.3% difference) in patients over 40.

We found that most patients presented on weekends (118, 40.4%) and between 6 pm and 4 am (161, 55.1%) (Figures 5, 6), consistent with other studies that have found weekend and night presentations the most common [6,7,14,19,20]. Despite our initial suspicion that there would be a greater proportion of patients endorsing alcohol use on weekends, we observed no such difference in the current study.

A total of 39 (13.4%) patients in this study endorsed alcohol use before their e-scooter accident (Figure 3). We suspect this to be underreported, as alcohol use was only flagged if noted specifically in the ED narrative. Alcohol endorsers in this study were twice as likely to suffer a head injury than non-endorsers, more than twice as likely to have emergent acuity, and 24.0% more likely to present via EMS (Table 1). Other studies have found that alcohol intoxication was associated with more severe injuries from e-scooter accidents [13,15], and Zube et al. (2022) found that e-scooter driving performance was impaired even at low blood alcohol levels [24]. We believe that law enforcement agencies should be aware of these dangers and act accordingly to deter intoxicated e-scooter riding.

This study found that a large proportion of patients arrived at the ED via ambulance (119, 40.8%) (Figure 7). Although difficult to compare across studies due to region and hospital-specific differences, this was higher than reported in some previous studies such as Beck et al. (2019) (7%), Badeau et al. (2019) (24%), and Siow et al. (2020) (36.9%) [7,8,13]. These numbers represent a significant burden on the city’s EMS system that is likely to increase as e-scooters become a more prevalent form of travel. EMS agencies both in Tampa and in other cities with a high burden of e-scooter injuries may see it fit to advise their personnel on the management of such injuries and be prepared for them to increase in volume.

We also found a significantly higher proportion of hospital admissions (92, 31.5%) (Figure 9) than reported in a systematic review of studies on e-scooter injuries by Toofany et al. (2021) (mean 14%) [5]. However, higher admission proportions have been observed in other studies such as Mayhew et al. (2019) (40%) and Siow et al. (2020) (36.7%) [3,13]. Regardless of region-specific differences, the hospital burden of e-scooter injuries is by no means limited to the ED, and many specialties may be recruited in the management of these patients.

The significant EMS utilization and admission rates in Tampa for such injuries are highly notable. These findings, along with the high rates of urgent (204, 69.9%) and emergent (51, 17.5%) acuity ED intakes (Figure 8), may point to the presence of more serious accidents in our city, although more research is needed to establish such causation. Although injury severity has been inconsistently reported in the literature [5], severe e-scooter injuries are common [3,5-8,11,13], and the U.S. National Transportation Safety Board has reported at least 119 e-scooter or e-bike fatalities from 2017 to 2021 [21]. Two fatalities were observed in this study (Figure 9), and even more have been reported in the local news media [25,26].

The rapid increase in the prevalence of severe e-scooter injuries in Tampa is alarming and should alert lawmakers and the public to the dangers of e-scooter use. We believe that in general, and especially in cities with high EMS utilization, hospital burden, and fatalities associated with e-scooter injuries, lawmakers and e-scooter companies should strongly consider updating regulations to help maintain the safety of riders and pedestrians and prevent serious injuries and deaths.

One possible safety measure would be requiring the use of helmets for e-scooter riders. This study found an extremely low rate of helmet use (2.1%), consistent with the findings of several other studies [5,7,8,11-14]. Considering the high rate of head injuries reported in this study (40.8%) and others [3,5-8,10-12], helmet requirements on e-scooters may be a wise preventative measure. Cities such as Tel Aviv, Israel, where e-scooter injuries are common, have mandated shared e-scooters to be equipped with helmets and license plates, and e-scooter companies are limited in the number of e-scooters they are allowed to have in the city [27]. In contrast, e-scooter companies in the United States have made active efforts to reduce safety precautions, such as one company sponsoring a bill to get rid of helmet mandates in California [28]. While safe riding is largely the responsibility of the e-scooter rider, we believe e-scooter companies should be promoting safe practices and be responsible for ensuring that protective gear is available to their customers.

At the hospital and prehospital levels, it may become useful to create algorithms for the workup and management of patients presenting with e-scooter injuries. For example, given the widespread lack of helmet use among e-scooter riders, spinal precautions may be necessary until a spinal injury can be definitively ruled out in the ED. With the prevalence of upper and lower extremity injuries, stabilization of injured limbs in the field may prevent further deterioration during transport. If alcohol use or altered mental status is suspected, more caution may be warranted to rule out injuries that may not be obvious to the patient or provider. Upon ED intake, head CTs may assist in ruling out life-threatening trauma if a head injury is suspected.

Educational campaigns at the grade-school level may also play a role in reducing unsafe e-scooter riding practices, as many adolescents may use or begin using e-scooters as they become more prevalent. Elder et al. (2005) showed that school-based media campaigns are effective at reducing alcohol-related vehicle collisions [29]. We suggest that school systems and mass media campaigns incorporate information on the dangers of intoxicated e-scooter riding into the many preexisting programs aimed at preventing intoxicated driving.

Limitations

As a retrospective chart review, this study was limited by the availability of data collected through the EMRs of the patient sample. The most common CCs were MVC and fall, which do not provide any details on injury type and thus limited our ability to extrapolate insights regarding specific injuries. Due to such incompleteness of the injury data, injury severity scores were also not calculated. Flags for alcohol use, helmet use, altered mental status, and head injuries not listed as the CC were collected from the ED narrative, and were thus subjective and may provide an incomplete clinical picture. The majority of narratives did not document helmet or alcohol use, and thus they may be higher than found. Due to the nature of the operational report used to extract our sample from the EMR system, it is possible that e-scooter cases were missed if documented under an unrelated encounter code. Additionally, cases were excluded if there was any doubt that they were not true e-scooter accidents, furthering the possibility of missed cases.

## Conclusions

The introduction of a shared e-scooter rental program in Tampa has resulted in a new injury hazard that is growing in prevalence. E-scooter accidents in our study were characterized by high rates of head injuries, hospital admissions, and EMS transports. Additionally, alcohol endorsers showed increased rates of head injuries and EMS transports when compared to non-endorsers, suggesting a higher risk profile of e-scooter use in conjunction with alcohol intoxication. In general, helmet use was low, suggesting a significant lack of safety precautions among e-scooter riders. Our data highlight a growing public health concern and a multifactorial burden on the city’s healthcare system. Considering the rapid growth of shared e-scooters and their associated injuries, we believe that hospital and ED data should not be taken lightly in the context of future policies regarding e-scooters. We call upon lawmakers and e-scooter companies to strongly consider the emerging data on e-scooter risks and system burdens and be proactive in their efforts to prevent their associated morbidity and mortality.
